# Response of the modified GAFCHROMIC EBT2 radiochromic film to DC glow discharge plasma

**DOI:** 10.1038/s41598-024-52628-w

**Published:** 2024-01-27

**Authors:** Omar F. Farag, Omar M. Kotb, M. El Ghazaly, Naglaa M. EL-Sayed

**Affiliations:** Department of Physics, Faculty of Science, Zagazig University, PO 44519, Zagazig, Egypt

**Keywords:** Techniques and instrumentation, Physics, Plasma physics

## Abstract

The response of the modified GAFCHROMIC EBT2 radiochromic film to DC Oxygen glow discharge plasma was investigated using a flatbed scanner and an UV–Vis spectrophotometer. The film was modified by removing the polyester overlaminate, adhesive, and topcoat layers with a total thickness of 80 µm, and is now referred to as EBT2-M. The EBT2-M films were exposed to DC Oxygen plasma for different durations: 0, 0.5, 1, 2, 3, 4, 7, and 10 min. The exposed films exhibit coloration homogeneity with an average variation of (1.6 ± 0.3) × 10^−4^ pixel values/µm, irrespective of the applied exposure time. The pixel values of the red-and-green channels and weighted grayscale images decreased exponentially with different sensitivity amounts to $\sim$ 39.67, 49.69, and 42.11 min^−1^, respectively, as the exposure time increased. The two absorption peaks at 580 ± 4 nm and 632 ± 4 nm in the UV–Vis absorption spectra of the exposed GAFCHROMIC EBT2-M radiochromic films are increasing with increasing exposure time up to 4 min, thereafter saturated for prolonged exposure time. The integrated absorbance in the range from 400 to 700 nm is linearly correlated with the exposure time. The indirect and direct optical energy band gaps and Urbach energy of the modified GAFCHROMIC EBT2 film are weakly correlated with the exposure time. These findings suggest the utilization of the modified GAFCHROMIC EBT2 radiochromic film as a novel and simple technique for plasma diagnostics.

## Introduction

Self-developing GAFCHROMIC EBT2 radiochromic film is intensively applied as a two dimensional radiation dosimeter for photons and charged particles in radiation therapy^1^ and references therein. GAFCHROMIC EBT2 film is characterized by low energy dependence over the range from about 0.05 MeV to some MeV for photons, tissue equivalent material with an effective atomic number of 6.84, and less sensitivity to the interior room light and temperature^2–5^. The radiation energy deposition in the GAFCHROMIC EBT2 film gives rise to 1,4 polymerization of the diacetylene monomers that form the EBT2 active layer, and accordingly, the coloration of the EBT2 film increases^1,6–8^. Nevertheless, EBT2 film could be modified by carefully removing the polyester overlaminate, adhesive, and topcoat layers with a total thickness of 80 µm to permit registration of low-energy charged particles and ions^9^. It is worthy of mention that such modifications have also been applied in other different studies^9–11^.

The glow discharge plasma, which is a form of low temperature plasma (LTP)^12,13^ is widely applied in medical applications^14–22^ as well as materials processing^23–29^. The glow discharge plasma consists of positive ions, electrons, and reactive species, including radicals and excited atoms, as well as UV photons^12,13^. In the surface modification of materials, four main processes may occur; ablation, polymerization, functionalization, and crosslinking^25,30^. These processes are controlled by many factors of the treatment conditions, including the gas type and pressure, the power applied, and the time of exposure, in addition to the type of treated material^29,31^. For the GAFCHROMIC EBT2 radiochromic film, LTP induces polymerization of the diacetylene monomer and gives rise to the coloration of the film. LTP surface modification is limited to a depth of few hundred Angstroms, leaving the bulk material properties unchanged^32^, in addition to a uniformity over large planar areas^33^.

The objective of the current work is to investigate the response of modified GAFCHROMIC EBT2 films (EBT2-M) upon exposure to DC glow discharge oxygen plasma. The correlation between the pixel values of each color channel of GAFCHROMIC EBT2-M films and the exposure time is studied. The dynamic range and sensitivity of each color channel will be determined. The induced modifications in the optical properties of the GAFCHROMIC EBT2-M films exposed to DC oxygen plasma will be reported. The two characteristic absorption peaks at the wavelengths of 580 ± 4 nm and 632 ± 4 nm are studied as a function of exposure time to DC oxygen plasma. In addition, the indirect and direct optical energy band gaps and Urbach energy of the GAFCHROMIC EBT2-M films exposed to DC Oxygen plasma will be reported.

## Materials and methods

**Figure 1:**
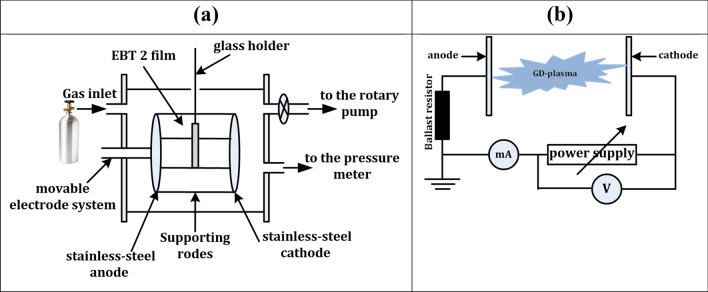
(**a**) A block diagram of the DC glow discharge plasma unit. (**b**) Electrical circuit. The modified GAFCHROMIC EBT2 radiochromic film is located parallel to the two electrodes in front of the cathode.

The plasma unit consists of a Pyrex cylindrical tube 18 cm long and 13 cm in diameter, flanged at both ends with an aluminum plate. Two stainless-steel circular electrodes of 5 cm diameter and 7 cm spacing are connected to a DC power supply. The tube was evacuated to a base pressure of 10^–3^ Torr by using a rotary pump (Edwards's vacuum pump, model ED 200). The tube was fed with O_2_, as a working gas, at a constant flow rate through a controllable needle valve. The plasma gas pressure amounted to 0.4 Torr. The discharge current and voltage applied are 7 mA and 500 Volt, respectively, corresponding to a power of 3.5 W and kept constant during the course of the measurements. A block diagram of the DC glow discharge plasma unit, which contains all components and their positions, is illustrated in Fig. 1.

The low-temperature plasma has a short penetration depth in polymer-based materials in the range of a few hundred Angstroms^16,30–34^; therefore, it is completely absorbed before the active layer. Accordingly, GAFCHROMIC EBT2 radiochromic film (Lot No. A06281102B) was modified to allow the low-energy plasma species to react with the EBT2 active layer. Firstly, the GAFCHROMIC EBT2 film was cut into different rectangular samples, each with an area of 1 × 2 cm^2^. At an angle of the rectangular sample, a fine needle was first used under an optical microscope with a magnification of 60X to separate a small part of the polyester overlaminate, adhesive, and topcoat layers with a total thickness of 80 µm. Thereafter, gently and carefully, we continued to remove the rest of the mentioned layers by hand. The modified film was inspected with an optical microscope of magnification 600X, which ensured the homogeneity of the modified film, while a digital micrometer was used to measure the film thickness, which amounts to 204 ± 1 µm, which is equal to the thickness of both the polyester substrate (175 µm) and the active layer (30 µm), which proves the removal of the topcoat layer. The GAFCHROMIC EBT2-M samples are fixed on a glass holder, thereafter, the films are inserted into the discharge tube and positioned at a distance of about 2 cm from the cathode surface (at the edge of the negative glow region of the glow discharge, see Fig. 1).

GAFCHROMIC EBT2-M films were exposed to DC Oxygen plasma for 0.5, 1, 2, 3, 4, 7, and 10 min, all measurements were carried out one day after exposure to minimize the post-exposure effect^1,6–8^. The films were scanned using a Canon CanoScan (Model LiDE 110) flatbed scanner in reflection mode^35^, and the images were saved in tif format. The scanner resolution amounts to 600 dpi 24-bit, 8-bit for each RGB color scale^36^. All films are read out from the modified side in portrait orientation. Free software code Image-J was used to analyze the images^37^. During scanning, all samples are oriented together to compensate for the polarization of the GAFCHROMIC films. All films are marked with a permanent pen to assure they are placed with the same orientation. The samples to be scanned were located in the center of the scanner bed. The UV–Vis absorption spectra were measured using a spectrophotometer (Model Spectro dual split beam, UVS-2700) at wavelengths in a range (400–700 nm)^38^.

**Figure 2:**
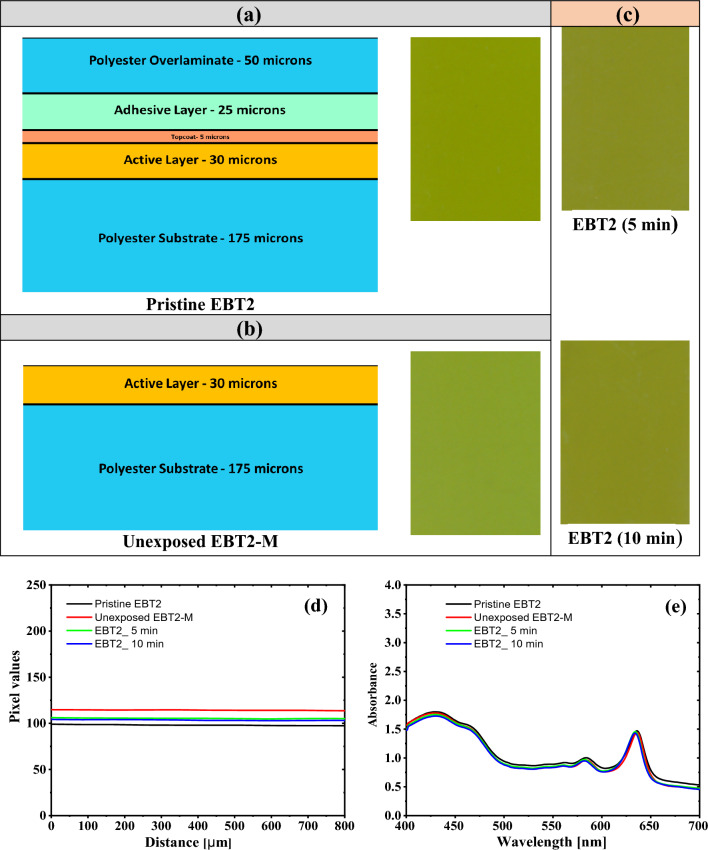
(**a**) Structure of the GAFCHROMIC EBT2 film and the corresponding image scanned by Cannon CanoScan (Model LiDE 110) flatbed scanner. (**b**) Modified GAFCHROMIC film (EBT2-M) by removing the polyester overlaminate, adhesive, and topcoat layers with a total thickness of of 80 μm, and its corresponding scanned image. (**c**) GAFCHROMIC EBT2 films exposed to DC Oxygen plasma for 5 and 10 min. (**d**) The projected line profile intensities of each image in a, b, and c. (**e**) The corresponding UV–Vis absorption spectra.

Figure 2a illustrates the structure of the GAFCHROMIC EBT2 film^39^ and the corresponding image scanned using a Cannon CanoScan (Model LiDE 110) flatbed scanner. Firstly, GAFCHROMIC EBT2 films were exposed to DC Oxygen plasma for 5 and 10 min to assess the accessibility of plasma species to reach and react with the EBT2 active layer. One can recognize that the color of the GAFCHROMIC EBT2 film exposed to DC Oxygen plasma, as indicated in Fig. 2c, is slightly darker than the pristine EBT2 film shown in Fig. 2a. However, the darkness (optical density) increment is attributed to the UV plasma photons that can easily penetrate both the polyester overlaminate-50 µm and adhesive layer-25 µm.

Apart from UV photons, all the plasma species are in the range of a few hundred Angstroms and completely absorbed within the polyester overlaminate-50 µm and adhesive layer-25 µm^16,30–34^. Accordingly, the removal of the polyester overlaminate layer of 50 μm, the adhesive layer of 25 μm, and topcoat layer of 5 μm, as indicated in Fig. 2b, allows the plasma species to reach and interact with the EBT2 active layer. The homogeneity of the GAFCHROMIC EBT2-M film was checked by a cannon canoscan flatbed scanner. The resulted image of the modified EBT2 film shows a high degree of homogeneity without any damage after removing the polyester overlaminate, adhesive, and topcoat layers, as shown in Fig. 2d. For quantitative analysis, the line profile for each image was produced by the vertical projection of each image pixel by pixel (see Fig. 2d). The line profile regression amounts to (1.6 ± 0.4) × 10^−4^, (2.1 ± 0.2) × 10^−4^, (1.6 ± 0.3) × 10^−4^, and (2.2 ± 0.3) × 10^−4^ for GAFCHROMIC EBT2, GAFCHROMIC EBT2-M, and EBT2 exposed to 5 min and 10 min in their respective orders, showing a high degree of homogeneity. It is worth mentioning that the pixel values of the GAFCHROMIC EBT2-M amount to 114 ± 2, while the grey level of pristine GAFCHROMIC EBT2 is 99 ± 2, which gives rise to an increase in the dynamic range by a factor of 15.15%.

UV–Vis absorption spectra of GAFCHROMIC EBT2, GAFCHROMIC EBT2-M, and EBT2 films exposed for 5 min and 10 min are depicted in Fig. 2e. The absorbance at the two characteristic peaks of 580 ± 4 nm and 632 ± 4 nm is decreasing from 1.003 and 1.470 of EBT2 film to 0.965 and 1.442 of EBT2-M, respectively, indicating an improvement in the transparency of EBT2-M film. For GAFCHROMIC EBT2 films exposed to DC oxygen plasma for 5 min and 10 min, the absorbance at 580 ± 4 is 0.943 and 0.966, while at 632 ± 4 nm it is 1.432 and 1.456, respectively.

## Results and discussion

In principle, for dose mapping in radiotherapy, a flatbed scanner in reflection or transmitted mode is applied to read out GAFCHROMIC films, while a UV–Vis spectrophotometer with the scanner could be applied for radiation dosimetry.

### RGB images by Flatbed scanner

**Figure 3:**
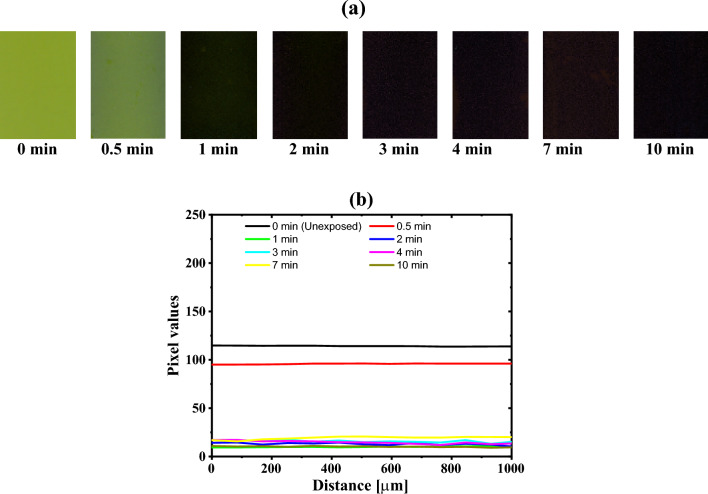
(**a**) Images of the GAFCHROMIC EBT2-M radiochromic films exposed to DC Oxygen plasma for different durations ranging from 0 to 10 min that are scanned using Canon CanoScan (Model LiDE 110) flatbed scanner. The area of each image is 25,000 × 17,700 μm^2^. (**b**) The projected line profile intensities of each image.

The GAFCHROMIC EBT2-M radiochromic films were exposed to DC Oxygen plasma, with a pressure of 0.4 Torr and a discharge power of approximately 3.5 W, for different exposure time: 0, 0.5, 1, 2, 3, 4, 7, and 10 min. One day after exposure, radiochromic films were digitized using a Cannon CanoScan (Model LiDE 110) flatbed scanner and saved in tif format^35^. Subsequently, Image-J free code software was used to analyze the images^37^, as illustrated in Fig. 3a. The area of each image is 25,000 × 17,700 μm^2^. The irradiated GAFCHROMIC EBT2-M films were scanned using the RGB mode. Triple color channels were quantitatively analyzed using the Image-J code. The RGB images are separated into three color channels; red, green, and blue. Furthermore, the RGB images are converted to weighted grayscale image, in which the effect of the blue channel is minimized, using the following equation^37^;

1 $$Weighted\;gray\;image = 0.{\text{299R}} + 0.{\text{587G}} + 0.{\text{114B}}.$$

As can be seen from Fig. 3a, the darkness of the GAFCHROMIC EBT2-M radiochromic films exposed to DC Oxygen plasma increases with the increase in the exposure time up to 3 min. For prolonged exposure time, the pixel values saturate, indicating that the GAFCHROMIC EBT2-M film has acquired sufficient energy under exposure to DC Oxygen plasma, inducing 1,4 polymerization of the diacetylene monomers in the EBT2 active layer.

Figure 3b shows the line profiles of unexposed GAFCHROMIC EBT2-M film and GAFCHROMIC EBT2-M film exposed to 10 min of DC Oxygen plasma with pixel values ranging from $\sim$ 115 to $\sim$ 11, respectively. Furthermore, the corresponding linear regression of the projected line profile intensities amounted to (2.1 ± 0.2) × 10^−4^ and (1.6 ± 0.3) × 10^−4^ Grey level/µm, respectively. This is proving the homogeneity of the GAFCHROMIC EBT2-M film, which is in good agreement with other works^7,9^.

**Figure 4:**
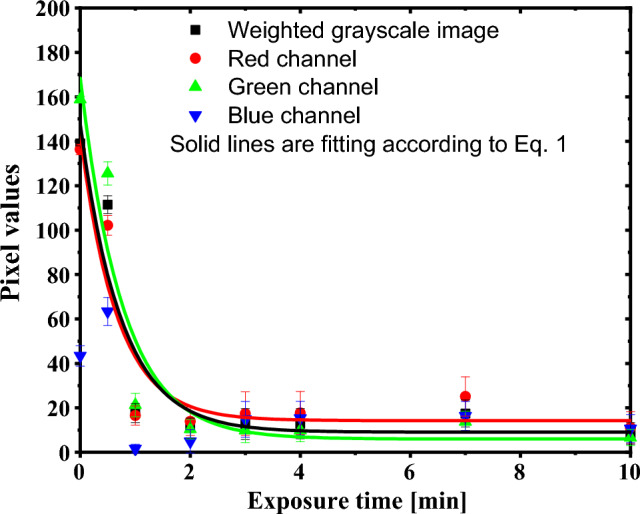
Color channel levels of the GAFCHROMIC EBT2-M films that were exposed to DC Oxygen plasma for different durations ranging from 0 to 10 min.

Triple color channels were quantitatively analyzed using the Image-J code. The RGB images are separated into three color channels; red, blue, and green, in addition to weighted grayscale image. Figure 4 displays the mean pixel values and associated standard deviation for GAFCHROMIC EBT2-M films exposed to DC Oxygen plasma and scrutinized as a function of exposure time to plasma. The data are fitted with three fitting parameters function:

2 $$G\left( {T_{Exp.} } \right) = a_{1} + a_{2} .e^{{( - a_{3} .T_{Exp.} )}} .$$

where a_1_ and a_2_, are dimensionless fitting parameters and a_3_ is the color decay constant expressed in pixel values/min. The fitting parameters are tabulated in Table 1.

**Table 1:** Fitting parameters in Eq. (2), of the color channels; grey, red, and green of the GAFCHROMIC EBT2-M films that were exposed to DC Oxygen plasma for different durations ranging from 0 to 10 min.

Color channel	${a}_{1}$	${a}_{2}$	${a}_{3}$ (Grey level min^−1^)	R
Grey	9.08 ± 10.11	138.4 ± 21.2	1.35 ± 0.49	0.94
Red	14.28 ± 8.98	128.7 ± 19.5	1.50 ± 0.54	0.95
Green	6.00 ± 10.95	162.4 ± 22.6	1.30 ± 0.43	0.95

The pixel values of the red-and-green channels, and weighted grayscale image of the GAFCHROMIC EBT2-M films exposed to DC Oxygen plasma decrease exponentially with the increase in exposure time, as shown in Fig. 4. This indicates a high sensitivity at short exposure time up to 3 min and diminishes at prolonged exposure. The sensitivity of the red, green, and weighted grayscale channels amounts to $\sim$ 39.67, 49.69, and 42.11 min^−1^, respectively. The dynamic range of the GAFCHROMIC EBT2-M films, the difference between the maximum and minimum measurable pixel values of the color channel, was also obtained from Fig. 4 and equals to 119 ± 7, 149 ± 4, 126 ± 5, and 28 ± 3 pixel values for red, green, grey, and blue, in their respective orders. For pristine GAFCHROMIC EBT2-M film, the green channel has the highest pixel values of 159 ± 1, thereafter the grey channel has a value of 139 ± 1, followed by the red channel with a value of 136 ± 2. Meanwhile, the blue channel has an insignificant value of 43 ± 5, indicating a poor response to DC Oxygen plasma. This is because of the yellow marker dye that is included in the EBT2 active layer, which absorbs the blue light without inducing a considerable contribution to the polymerization of the film. Such an observation is in agreement with the literature studies concerned with the response of GAFCHROMIC EBT2 and EBT3 to other types of radiation^1,6–8^. The combined image of radiochromic film is deteriorated by the presence of the blue channel; therefore, scanning the film using a monochromatic wavelength of the red and green channels would give rise to a higher dynamic range and sensitivity of the film.

### Optical properties of the GAFCHROMIC EBT2-M film

**Figure 5:**
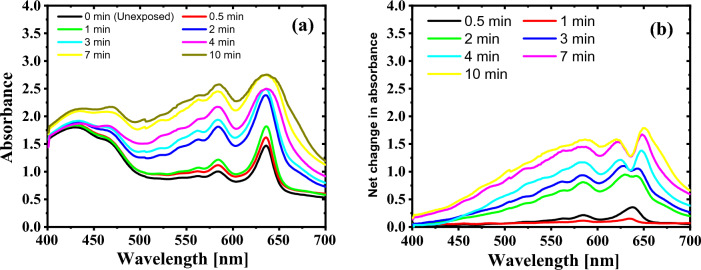
(**a**) UV–Vis absorption spectra for the GAFCHROMIC EBT2-M films exposed to DC Oxygen plasma and (**b**) the net absorbance spectra as a function of wavelength, for different exposure time. The wavelength is ranging from 400 to 700 nm.

The induced optical modifications of the GAFCHROMIC EBT2 films upon exposure to DC Oxygen plasma could be quantitatively analyzed by UV–Vis spectroscopy^7,9,40^. UV–Vis absorption spectra for the pristine GAFCHROMIC EBT2-M film and the GAFCHROMIC EBT2-M films exposed to DC Oxygen plasma were measured in the wavelength range of 400–700 nm as shown in Fig. 5a. The two characteristic peaks are observed in the absorption spectra at the wavelengths of 580 ± 4 nm and 632 ± 4 nm for the GAFCHROMIC films. These two peaks are attributed to the electronic transition between the highest occupied molecular orbitals (HOMO) and the lowest unoccupied molecular orbitals (LUMO) in the active dye layer of EBT2-M film^6,9^. The absorbance intensity at all wavelengths increased as the exposure time to plasma increased up to 4 min; thereafter, it tended to saturate.

Figure 5b shows the net absorbance spectra of the GAFCHROMIC EBT2-M films exposed to DC Oxygen plasma for different exposure time, which are produced by subtraction of the pristine film from the exposed GAFCHROMIC EBT2-M films. The net absorption between wavelengths of 600 and 650 nm became more complicated with the increase in exposure time that is ascribed to the saturation of the absorption starting from 4 min, which is associated with flatness in the characteristic peak at 632 ± 4 nm.

The increase in the absorbance of the GAFCHROMIC EBT2-M films is attributed to the plasma-induced 1,4-polymerization of diacetylene monomer in the active layer of the films. This produces a linear long-chain polymer (polyPCDA) with alternating double and triple carbon–carbon bonds in the backbone. The diacetylene monomer is of low absorbance in the visible part of the spectrum with two characteristic peaks at 580 ± 4 nm (green light) and 632 ± 4 nm (red light), while the darkened polydiacetylene absorbs light due to the extensive delocalization of π-orbital electrons along the polymer backbone^41^. The saturation of the GAFCHROMIC EBT2-M films absorbance at prolonged exposure time is attributed to the complete polymerization of the diacetylene monomers in the active layer. The obtained results reveal that the absorbance of the GAFCHROMIC EBT2-M films exposed to DC Oxygen plasma is strongly correlated with the exposure time, showing the possibility of using the GAFCHROMIC EBT2-M film in low temperature plasma investigations^42^.

**Figure 6:**
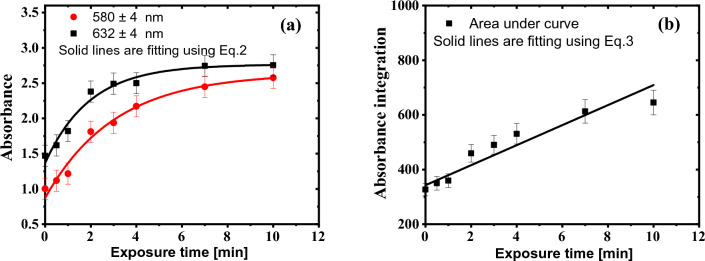
(**a**) The response curves of the GAFCHROMIC EBT2-M films a function of exposure time to DC Oxygen plasma at the two characteristic absorption peaks of wavelengths 580 ± 4 nm and 632 ± 4 nm. (**b**) The corresponding integrated absorbance in the range of wavelengths from 400 to 700 nm.

Figure 6a describes the response of the GAFCHROMIC EBT2-M films as a function of exposure time to DC Oxygen plasma at the two characteristic absorption peaks of wavelengths 580 ± 4 nm and 632 ± 4 nm, where the data are fitted using the following equation:

3 $$A\left( {T_{exp.} } \right) = b_{1} - b_{2} e^{{b_{3} T_{exp.} }}$$

where b_1_, b_2_, and b_3_ are fitting parameters and their values are illustrated in Table 2, b3 has the unit of min^−1^. The sensitivity of the GAFCHROMIC EBT2-M films to DC Oxygen plasma is calculated by the first order differentiation of Eq. (3) with respect to the exposure time.

**Table 2:** Fitting parameters according to Eq. (3) of the absorbance of the GAFCHROMIC EBT2-M films exposed to DC Oxygen plasma at the two peaks of wavelengths 580 ± 4 nm and 632 ± 4 nm for different durations ranging from 0 to 10 min.

Peak position (nm)	$b$_1_	${b}_{2}$	${b}_{3} \,{{\text{min}}}^{-1}$	R
580 ± 4	2.64 ± 0.05	1.77 ± 0.09	0.328 ± 0.038	0.99
632 ± 4	2.76 ± 0.04	1.40 ± 0.16	0.513 ± 0.085	0.99

**Table 3:** Indirect and direct energy band gaps, Urbach's energy values for GAFCHROMIC EBT2-M films exposed to DC Oxygen plasma for different exposure time.

T_exp_ (min)	Indirect E_g_ (eV)	Direct E_g_ (eV)	${\text{U}}$ (eV)	$\Delta {\text{U}}$ (eV)
0	1.82 ± 0.01	1.90 ± 0.01	0.079	0.004
0.5	1.82 ± 0.01	1.90 ± 0.01	0.083	0.005
1	1.82 ± 0.01	1.90 ± 0.01	0.065	0.003
2	1.76 ± 0.01	1.88 ± 0.01	0.076	0.001
3	1.75 ± 0.01	1.88 ± 0.01	0.082	0.001
4	1.71 ± 0.01	1.85 ± 0.01	0.110	0.004
7	1.68 ± 0.01	1.84 ± 0.01	0.141	0.006
10	1.61 ± 0.01	1.81 ± 0.01	0.227	0.012

The absorbance at the two characteristic peaks at the wavelengths 580 ± 4 nm and 632 ± 4 nm increases according to Eq. (3) with plasma exposure time up to 4 min; thereafter, it saturates. The energetic active plasma species interact with the active layer (diacetylene monomer) in the GAFCHROMIC EBT2-M films, inducing the radical polymerization reaction and producing a higher optical density (darkness) of polydiacetylene. At prolonged exposure time to DC Oxygen plasma, all the diacetylene monomers in the active layer are transformed into polymers, and the concentration of the newly formed polymer becomes constant. As a result, the absorbance saturates.

The integrated absorbance in the range of wavelengths from 400 to 700 nm is linearly correlated with the exposure time to DC Oxygen plasma, as shown in Fig. 6b. The data are fitted with a linear equation as following:

4 $$Area\left( {T_{exp.} } \right) = c_{1} + c_{2} T_{exp.}$$

where c_1_ and c_2_ are fitting parameters and their values are 342.70 ± 15.53, 36.64 ± 4.57 min^−1^, respectively, with an R-square value of 0.91. The fitting parameter c_2_ represents the sensitivity in min^−1^ of the GAFCHROMIC EBT2-M films to plasma. Despite the higher degree of linearity of the integrated absorbance of the GAFCHROMIC EBT2-M films exposed to DC Oxygen plasma, a poor sensitivity was observed that amounts to 36.64 ± 4.57 min^−1^. In other words, using the integrated absorbance, the dynamic range is extended to measure the effect of plasma from 0 to 10 min on the cost of sensitivity.

### Determination of optical energy band gaps and Urbach's energy

The coloration of the GAFCHROMIC EBT2-M films proves the chemical changes induced by the energy deposition from the DC glow discharge plasma. This is associated with a change in the optical energy gaps and Urbach’s energy, which can be calculated from UV–Vis absorption spectra. The optical absorption coefficient, α(hν) is calculated by using the following relation^43,44^:

5 $$\alpha \left( {h\nu } \right) = 2.303 \frac{A\left( \lambda \right) }{l} .$$

where *A* and *l* are the absorbance and the thickness of the film, respectively.

**Figure 7:**
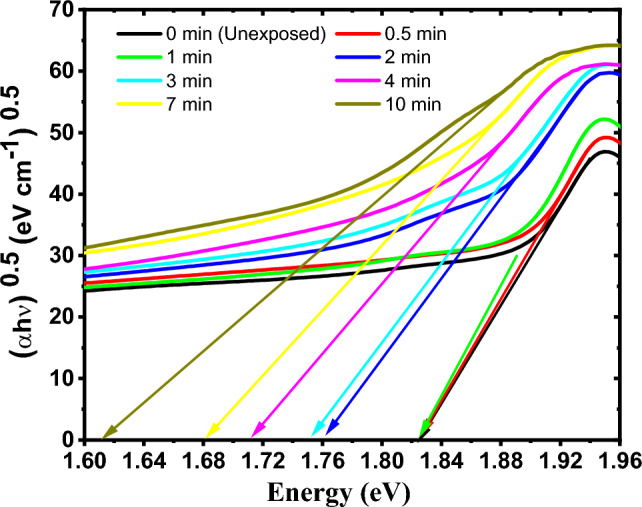
The indirect band gap in the GAFCHROMIC EBT2-M films exposed to DC Oxygen plasma for different exposure time.

**Figure 8:**
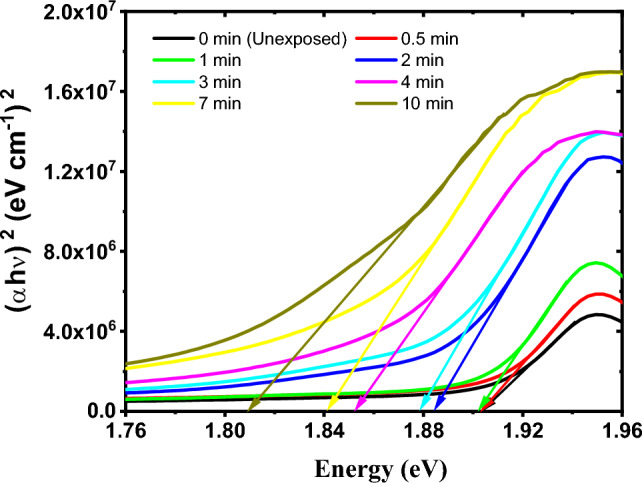
The direct band gap in the GAFCHROMIC EBT2-M films exposed to DC Oxygen plasma for different exposure time.

The indirect and direct energy band gaps of the GAFCHROMIC EBT2-M films exposed to DC Oxygen plasma were determined by plotting (*α*hν)^1/2^ and (*α*hν)^2^ versus the photon energy (hν) according to Tauc’s equation, respectively^31,45^. The intersection of the extension of the straight part of the curves with the energy (hν) axis produces the indirect and direct energy band gaps values, respectively, as illustrated in Figs. 7 and 8.

**Figure 9:**
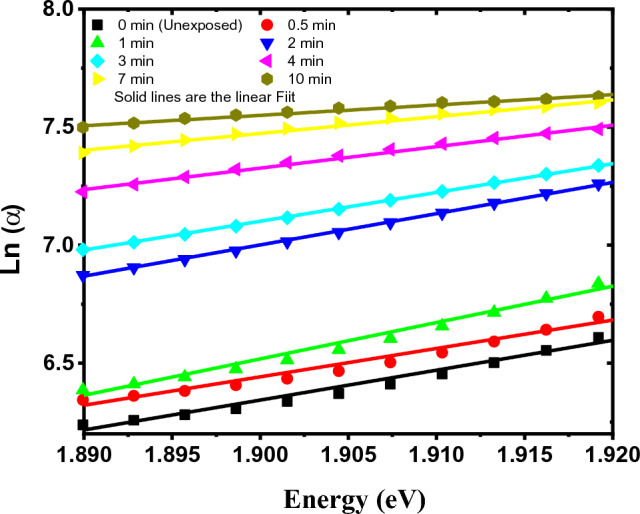
The natural logarithm of the absorption coefficient (α) versus photon energy for the GAFCHROMIC EBT2-M films exposed to DC Oxygen plasma for different exposure time.

The values of the Urbach energy (U) or the band tail width were determined by using the Urbach rule from the reciprocal of the slope of the logarithmic α(hν) against the photon energy (hν)^31,45^ as shown in Fig. 9. The Urbach energy uncertainty ($\Delta U)$ is estimated in terms of the slope (S) and its uncertainty $(\Delta S)$ using the following equation:

6 $$\Delta {\text{U}} = \sqrt {\left( {\frac{\partial U}{{\partial S}}\Delta S} \right)^{2} } = \frac{\Delta S}{{S^{2} }}{ }$$

The values of the optical energy band gaps; E_g_, Urbach energy (U), and the corresponding Urbach energy uncertainty ($\Delta U)$ calculated by Eq. (6), for the GAFCHROMIC EBT2-M films upon exposure to DC Oxygen plasma for different exposure time are listed in Table 3.

From Table 3, it can be seen that exposing the GAFCHROMIC EBT2-M films to DC glow discharge plasma for a short exposure time up to 1 min doesn't influence the values of the indirect and direct optical energy band gaps of the exposed films. While the increase in exposure time from 1 min. to 10 min. decreases the values of the indirect and direct optical energy band gaps of the films. The decrease in the values of E_g_ can be attributed to the development of new photochemical processes that create the trap levels between the HOMO and LUMO energy states, which makes the lower energy transitions feasible and consequently results in the decrease in the optical energy band gaps^46^. Within the exposure time range, the change in the indirect optical energy gap is higher than that in the direct one.

On the other hand, the obtained values for Urbach energy (E_u_) of the GAFCHROMIC EBT2-M films exposed to DC Oxygen plasma fluctuate up to 1 min; thereafter, they increase with the increase in exposure time. The increase in the values of Urbach energy (E_u_) for prolonged durations may be attributed to the degradation of the polydiacetylene by the deposited energy from active plasma species^24,47^.

## Conclusion

The response of the modified GAFCHROMIC EBT2 radiochromic film to DC glow discharge plasma was investigated for the first time. The modified GAFCHROMIC EBT2 film (EBT2-M) exposed to DC Oxygen plasma for different durations exhibited high coloration homogeneity even for the maximum exposure time of 10 min with an average variation of (1.6 ± 0.3) × 10^−4^ pixel values/µm. The pixel values of the red and green channels, and weighted greyscale image were decreased exponentially, where the green channel has the highest dynamic range and sensitivity. The two characteristic absorption peaks at 580 ± 4 nm and 632 ± 4 nm are increasing with the increase in the exposure time up to 4 min, thereafter saturated for prolonged exposure time. The integrated absorbance in the range from 400 to 700 nm is a linear function of the exposure time. The indirect and direct optical energy band gaps and Urbach energy of the GAFCHROMIC EBT2-M films exposed to DC Oxygen plasma are weakly correlated with the exposure time. Accordingly, the modified GAFCHROMIC EBT2 radiochromic film may be used as a novel and simple technique for plasma diagnostics. However, deep investigations of the correlation between the radiochromic film response and the widespread parameters of plasma source are required. These plasma parameters include gas type, gas pressure, discharge voltage, current, and plasma treatment time.

## Data Availability

All data generated or analyzed during this study are included in this published article.
